# Supplementary material for: Cytomegalovirus reactivation in mechanically ventilated patients with or without SARS-CoV-2 infection: A retrospective cohort study
Source: PLoS One. 2025 Oct 27;20(10):e0328494. doi: 10.1371/journal.pone.0328494 (PMC12558532; doi:10.1371/journal.pone.0328494)
Supplement: S1 Table — Stepwise downward selection method forcing the variable « SARS-CoV-2 positive ». (DOCX) [file pone.0328494.s001.docx]

| **Table S1. Factors associated with CMV reactivation in the sub-population with known seropositive CMV status (n=129).** Stepwise downward selection method forcing the variable « SARS-CoV-2 positive ». | | | | | | |
| --- | --- | --- | --- | --- | --- | --- |
|  | **Unadjusted** | | | **Adjusted** | | |
|  | **SHR** | **CI at 95%** | **P-value** | **SHR** | **CI at 95%** | **P-value** |
| SARS-CoV-2 positive | 1.68 | 1.03-2.72 | 0.04 | 1.08 | 0.61-1.93 | 0.78 |
| Methylprednisolone | 2.73 | 1.71-4.35 | <0.001 | 2.76 | 1.53-4.96 | <0.001 |
| Time from hospital admission to intubation | 1.01 | 0.99-1.03 | 0.37 | 1.02 | 0.99-1.04 | 0.06 |
| Dexamethasone | 1.41 | 0.89-2.24 | 0.15 |  |  |  |
| ARDS | 2.44 | 0.98-6.10 | 0.06 |  |  |  |
| ECMO | 2.19 | 1.41-3.40 | <0.001 |  |  |  |
| Blood transfusion | 2.6 | 1.44-4.96 | 0.002 |  |  |  |
| Prone positioning | 2.47 | 1.35-4.51 | 0.003 |  |  |  |
| SOFA | 1.02 | 0.96-1.09 | 0.57 |  |  |  |
| VAP occurence | 1.1 | 0.70-1.72 | 0.68 |  |  |  |
| *CMV = Cytomegalovirus; SHR=Sub-Hazard Ratio; CI=Confidence Interval; RR=Relative Risk; ARDS = Acute Respiratory Distress Syndrome; ECMO = Extra Corporeal Membrane Oxygenation; SOFA = Sequential Organ Failure Assessment; VAP = Ventilator Associated Pneumonia* | | | | | | |
