# Supplementary material for: Cytomegalovirus reactivation in mechanically ventilated patients with or without SARS-CoV-2 infection: A retrospective cohort study
Source: PLoS One. 2025 Oct 27;20(10):e0328494. doi: 10.1371/journal.pone.0328494 (PMC12558532; doi:10.1371/journal.pone.0328494)
Supplement: S2 Table — Adjusted and unadjusted analysis. (DOCX) [file pone.0328494.s002.docx]

| **Table S2. Risk factors for mortality in the first 60 days post intubation in the sub-population with known seropositive CMV status (n=129).** Adjusted and unadjusted analysis. | | | | | | |
| --- | --- | --- | --- | --- | --- | --- |
|  | **Unadjusted** | | | **Adjusted** | | |
|  | **HR** | **CI at 95%** | **P-value** | **HR** | **CI at 95%** | **P-value** |
| SARS-CoV-2 | 1.17 | 0.64-2.15 | 0.6 | 2.15 | 0.98-4.73 | 0.06 |
| CMV reactivation | 1.91 | 0.97-3.74 | 0.05 | 1.91 | 0.91-4.01 | 0.09 |
| Cancer | 2.22 | 1.02-4.83 | 0.05 | 1.32 | 0.47-3.70 | 0.6 |
| Alcohol consumption | 2.89 | 0.69-12.17 | 0.15 | 3.07 | 0.68-14.14 | 0.15 |
| Sepsis | 2.38 | 0.73-7.72 | 0.15 | 2.35 | 0.52-10.64 | 0.27 |
| VAP occurence | 0.74 | 0.40-1.35 | 0.32 | 0.75 | 0.40-1.43 | 0.38 |
| Age | 1.04 | 1.01-1.07 | 0.005 | 1.04 | 1.01-1.08 | 0.02 |
| SAPS 2 score | 1.04 | 1.01-1.07 | 0.005 | 1.04 | 1.02-1.07 | <0.001 |
| Time from hospital admission to intubation | 1.02 | 0.99-1.05 | 0.07 | 1.038 | 1.01-1.07 | 0.02 |
| *VAP = Ventilator Associated Pneumonia; SAPS 2=Simplified Acute Physiology Score 2; CMV = Cytomegalovirus; HR=Hazard Ratio; CI=Confidence Interval.*  ***NB : CMV reactivation was treated as a time dependent variable*** | | | | | | |
