# Supplementary material for: Cytomegalovirus reactivation in mechanically ventilated patients with or without SARS-CoV-2 infection: A retrospective cohort study
Source: PLoS One. 2025 Oct 27;20(10):e0328494. doi: 10.1371/journal.pone.0328494 (PMC12558532; doi:10.1371/journal.pone.0328494)
Supplement: Attachment — Submitted filename: Reviewing PONE -Point by point responses.docx [file pone.0328494.s003.docx]

PONE-D-25-24190

Cytomegalovirus reactivation in mechanically ventilated patients with or without SARS-CoV-2 infection: A comparative study.

PLOS ONE

Dear Dr. HRAIECH,

Thank you for submitting your manuscript to PLOS ONE. After careful consideration, we feel that it has merit but does not fully meet PLOS ONE’s publication criteria as it currently stands. Therefore, we invite you to submit a revised version of the manuscript that addresses the points raised during the review process.

Please clarify CMV testing criteria and rationale, improve methodological transparency, address the interpretation of controversial results, and place findings in the context of existing literature.

Please submit your revised manuscript by Sep 14 2025 11:59PM. If you will need more time than this to complete your revisions, please reply to this message or contact the journal office at [plosone@plos.org](mailto:plosone@plos.org). When you're ready to submit your revision, log on to [https://www.editorialmanager.com/pone/](https://urldefense.com/v3/__https:/www.editorialmanager.com/pone/__;!!JQ5agg!ZuTmpheA5EvGYYXLom0TNFeVPgbWmj4p3xQmpoNQifisfAMHACt_vpjaXYHJfXImfEasx8pSmyaQJw0fFk6A$) and select the 'Submissions Needing Revision' folder to locate your manuscript file.

Please include the following items when submitting your revised manuscript:

- A rebuttal letter that responds to each point raised by the academic editor and reviewer(s). You should upload this letter as a separate file labeled 'Response to Reviewers'.
- A marked-up copy of your manuscript that highlights changes made to the original version. You should upload this as a separate file labeled 'Revised Manuscript with Track Changes'.
- An unmarked version of your revised paper without tracked changes. You should upload this as a separate file labeled 'Manuscript'.

If you would like to make changes to your financial disclosure, please include your updated statement in your cover letter. Guidelines for resubmitting your figure files are available below the reviewer comments at the end of this letter.

If applicable, we recommend that you deposit your laboratory protocols in protocols.io to enhance the reproducibility of your results. Protocols.io assigns your protocol its own identifier (DOI) so that it can be cited independently in the future. For instructions see: [https://journals.plos.org/plosone/s/submission-guidelines#loc-laboratory-protocols](https://urldefense.com/v3/__https:/track.editorialmanager.com/CL0/https:*2F*2Fjournals.plos.org*2Fplosone*2Fs*2Fsubmission-guidelines*23loc-laboratory-protocols/1/010f01986054b280-82693657-4a3d-462a-8e7f-e1df07129cd1-000000/bD-ZMYqQ1Od8kGnCZD8JZ0byYqZqlmXh2R6k-QfqRbg=222__;JSUlJSUl!!JQ5agg!ZuTmpheA5EvGYYXLom0TNFeVPgbWmj4p3xQmpoNQifisfAMHACt_vpjaXYHJfXImfEasx8pSmyaQJ4le91KB$). Additionally, PLOS ONE offers an option for publishing peer-reviewed Lab Protocol articles, which describe protocols hosted on protocols.io. Read more information on sharing protocols at [https://plos.org/protocols?utm_medium=editorial-email&utm_source=authorletters&utm_campaign=protocols](https://urldefense.com/v3/__https:/track.editorialmanager.com/CL0/https:*2F*2Fplos.org*2Fprotocols*3Futm_medium=editorial-email*26utm_source=authorletters*26utm_campaign=protocols/1/010f01986054b280-82693657-4a3d-462a-8e7f-e1df07129cd1-000000/gPcaNbWyBSj0CskIGCTY0TUR67UeOHzqbjXGjIsLGCI=222__;JSUlJSUl!!JQ5agg!ZuTmpheA5EvGYYXLom0TNFeVPgbWmj4p3xQmpoNQifisfAMHACt_vpjaXYHJfXImfEasx8pSmyaQJ-hqQMBF$).

We look forward to receiving your revised manuscript.

Kind regards,

Glenda Canderan, PhD
Academic Editor
PLOS ONE

Journal Requirements:

When submitting your revision, we need you to address these additional requirements.

1. Please ensure that your manuscript meets PLOS ONE's style requirements, including those for file naming. The PLOS ONE style templates can be found at

[https://journals.plos.org/plosone/s/file?id=wjVg/PLOSOne_formatting_sample_main_body.pdf](https://urldefense.com/v3/__https:/journals.plos.org/plosone/s/file?id=wjVg*PLOSOne_formatting_sample_main_body.pdf__;Lw!!JQ5agg!ZuTmpheA5EvGYYXLom0TNFeVPgbWmj4p3xQmpoNQifisfAMHACt_vpjaXYHJfXImfEasx8pSmyaQJ5uU7CYI$) and

[https://journals.plos.org/plosone/s/file?id=ba62/PLOSOne_formatting_sample_title_authors_affiliations.pdf](https://urldefense.com/v3/__https:/track.editorialmanager.com/CL0/https:*2F*2Fjournals.plos.org*2Fplosone*2Fs*2Ffile*3Fid=ba62*2FPLOSOne_formatting_sample_title_authors_affiliations.pdf/1/010f01986054b280-82693657-4a3d-462a-8e7f-e1df07129cd1-000000/mE2MGDaLaTfdi5dDi3O8cRkGdMnp-nLJpVUjCtW26eI=222__;JSUlJSUlJQ!!JQ5agg!ZuTmpheA5EvGYYXLom0TNFeVPgbWmj4p3xQmpoNQifisfAMHACt_vpjaXYHJfXImfEasx8pSmyaQJ_Mqv3Ar$)

2. PLOS requires an ORCID iD for the corresponding author in Editorial Manager on papers submitted after December 6th, 2016. Please ensure that you have an ORCID iD and that it is validated in Editorial Manager. To do this, go to ‘Update my Information’ (in the upper left-hand corner of the main menu), and click on the Fetch/Validate link next to the ORCID field. This will take you to the ORCID site and allow you to create a new iD or authenticate a pre-existing iD in Editorial Manager.

3. We note that your Data Availability Statement is currently as follows: All relevant data are within the manuscript and its Supporting Information files

Please confirm at this time whether or not your submission contains all raw data required to replicate the results of your study. Authors must share the “minimal data set” for their submission. PLOS defines the minimal data set to consist of the data required to replicate all study findings reported in the article, as well as related metadata and methods ([https://journals.plos.org/plosone/s/data-availability#loc-minimal-data-set-definition](https://urldefense.com/v3/__https:/journals.plos.org/plosone/s/data-availability*loc-minimal-data-set-definition__;Iw!!JQ5agg!ZuTmpheA5EvGYYXLom0TNFeVPgbWmj4p3xQmpoNQifisfAMHACt_vpjaXYHJfXImfEasx8pSmyaQJ9i3n8IO$)).

For example, authors should submit the following data:

- The values behind the means, standard deviations and other measures reported;
- The values used to build graphs;
- The points extracted from images for analysis.

Authors do not need to submit their entire data set if only a portion of the data was used in the reported study.

If your submission does not contain these data, please either upload them as Supporting Information files or deposit them to a stable, public repository and provide us with the relevant URLs, DOIs, or accession numbers. For a list of recommended repositories, please see [https://journals.plos.org/plosone/s/recommended-repositories](https://urldefense.com/v3/__https:/journals.plos.org/plosone/s/recommended-repositories__;!!JQ5agg!ZuTmpheA5EvGYYXLom0TNFeVPgbWmj4p3xQmpoNQifisfAMHACt_vpjaXYHJfXImfEasx8pSmyaQJxlOGCGs$).

If there are ethical or legal restrictions on sharing a de-identified data set, please explain them in detail (e.g., data contain potentially sensitive information, data are owned by a third-party organization, etc.) and who has imposed them (e.g., an ethics committee). Please also provide contact information for a data access committee, ethics committee, or other institutional body to which data requests may be sent. If data are owned by a third party, please indicate how others may request data access.

4. If the reviewer comments include a recommendation to cite specific previously published works, please review and evaluate these publications to determine whether they are relevant and should be cited. There is no requirement to cite these works unless the editor has indicated otherwise. 


[Note: HTML markup is below. Please do not edit.]

Reviewers' comments:

Reviewer's Responses to Questions

**Comments to the Author**

1. Is the manuscript technically sound, and do the data support the conclusions?

The manuscript must describe a technically sound piece of scientific research with data that supports the conclusions. Experiments must have been conducted rigorously, with appropriate controls, replication, and sample sizes. The conclusions must be drawn appropriately based on the data presented.

Reviewer #1: Yes

Reviewer #2: Partly

Reviewer #3: Partly

2. Has the statistical analysis been performed appropriately and rigorously?

Reviewer #1: Yes

Reviewer #2: I Don't Know

Reviewer #3: No

3. Have the authors made all data underlying the findings in their manuscript fully available?

The [PLOS Data policy](https://urldefense.com/v3/__http:/track.editorialmanager.com/CL0/http:*2F*2Fwww.plosone.org*2Fstatic*2Fpolicies.action*23sharing/1/010f01986054b280-82693657-4a3d-462a-8e7f-e1df07129cd1-000000/bQ43M_UVsNt2JK7H-1WOfwqiLb6Ei9QurXWq12MX25I=222__;JSUlJSU!!JQ5agg!ZuTmpheA5EvGYYXLom0TNFeVPgbWmj4p3xQmpoNQifisfAMHACt_vpjaXYHJfXImfEasx8pSmyaQJx6pZbBS$) requires authors to make all data underlying the findings described in their manuscript fully available without restriction, with rare exception (please refer to the Data Availability Statement in the manuscript PDF file). The data should be provided as part of the manuscript or its supporting information, or deposited to a public repository. For example, in addition to summary statistics, the data points behind means, medians and variance measures should be available. If there are restrictions on publicly sharing data—e.g. participant privacy or use of data from a third party—those must be specified.

Reviewer #1: Yes

Reviewer #2: No

Reviewer #3: Yes

4. Is the manuscript presented in an intelligible fashion and written in standard English?

PLOS ONE does not copyedit accepted manuscripts, so the language in submitted articles must be clear, correct, and unambiguous. Any typographical or grammatical errors should be corrected at revision, so please note any specific errors here.

Reviewer #1: Yes

Reviewer #2: Yes

Reviewer #3: Yes

5. Review Comments to the Author

Please use the space provided to explain your answers to the questions above. You may also include additional comments for the author, including concerns about dual publication, research ethics, or publication ethics. (Please upload your review as an attachment if it exceeds 20,000 characters)

**Reviewer #1:**

The manuscript by Cannac et al. assesses the correlation between reactivation of cytomegalovirus (CMV) in SARS-CoV-2-infected patients versus non-SARS-CoV-2-infected patients and the effect on patient outcome. The manuscript is well-written and informative. There are a few suggested changes that would improve the manuscript.

Major comment:
**1) Reviewer’s comment:**

Several previous studies have analyzed CMV reactivation in SARS-CoV-2 patients with mechanical ventilation; it is not clearly stated in the manuscript how the current study adds to or differs from these studies. There should be more information about this in the introduction and especially the discussion.

**Authors’ response:** we agree with the reviewer’s comment. We performed a careful re-analysis of the literature data available in this field. Several studies have limited effectives (under 100 patients) and their results, both on the incidence of CMV reactivation in SARS-CoV-2 patients and its impact on mortality are conflicting. Using competing risk methods, we did not confirm an association between SARS-CoV2 and CMV but we showed a negative effect on mortality of viral co-infection. The main originality of our cohort, apart from its size, stays in the fact that we put in light the role of methylprednisolone (and not dexamethasone) as a risk factor for CMV reactivation and a lower mortality in patients that were treated with ganciclovir. We placed our findings in the context of existing literature and what our study adds to the field in the introduction but especially in the discussion sections.

Minor comment:
**1) Reviewer’s comment:**

Lines 54-56 is a bit confusing and should be restated.

**Authors ‘response:**

This sentence has been reworded.

We’d like to thank the reviewer for his helpful comments

**Reviewer #2:**

Cytomegalovirus reactivation in mechanically ventilated patients with or without SARS-CoV-2 infection: A comparative study
This manuscript presents a monocentric retrospective cohort study comparing the incidence, risk factors, and outcomes of cytomegalovirus (CMV) reactivation in mechanically ventilated patients with and without SARS-CoV-2 infection. The study includes 330 patients, the authors used competing risk analysis (Fine & Gray model) and Cox regression to estimate the probability of CMV reactivation.
The authors report an association of treatment with methylprednisolone but not of SARS-CoV-2 infection with CMV reactivation . Moreover, among patients presenting a CMV reactivation, the administration of ganciclovir was associated with lower mortality.

**Reviewer’s comment:**
• The authors state, that all data are reported to be available without restriction, however, the submitted manuscript does not contain primary data.

**Authors ‘response:**

Primary data have been uploaded as Supporting Information files.

**Reviewer’s comment:**
• Overall, the retrospective nature of the study limits causal inference and increases the risk of selection and information bias. Although this limitation is somewhat acknowledged in the discussion, the authors draw unsupported conclusions from the data presented.

**Authors ‘response:**

We agree with the reviewer that the retrospective design of the study is a weakness and have stated it as the first limit as noted by the reviewer. Considering this, we have described our results in a careful manner and only presented the conclusions supported by strong statistical methods.

**Reviewer’s comment:**

The indication for CMV testing remains somewhat dubious (routine in patients after 4 days MV? Why?) and I would suspect that the test was ordered in patients not responding to treatment in timely manner, implying an important selection of patients with higher mortality.

**Authors ‘response:**

We acknowledge that the routine diagnosis of CMV reactivation in ICU patients is not consensual. Moreover, our description of CMV screening was confusing as we declared that there was no protocol for CMV screening. This statement concerned only respiratory tract CMV screening but not blood screening. We apologize for this omission and imprecision in the methods section.

In fact, during the study period, patients under IMV for at least 4 days were supposed to be screened twice weekly for CMV reactivation with PCR on whole blood for as long as they remained under IMV. This has become part of routine care in our ICU since a randomized controlled trial investigating the effect of preemptive ganciclovir in ICU patients with CMV reactivation (doi.org/10.1186/s13613-020-00793-2). In this trial, patients had a routine screening of CMV reactivation from the 4^th^ day of MV. This delay was determined from previous studies showing that CMV reactivation before this time point was rarely described and that CMV reactivation more frequently occurred after this time point (see response lower). Since this trial, we kept on practicing CMV routine screening in patients undergoing at least 4 days of MV as part of routine care, even though, as for all protocols of care, the observance was not perfect. This screening is therefore not correlated with patients’ severity.

Conversely, there was no protocol for CMV screening in LRT which was decided by clinicians according to patient’s evolution.

This has been added in the methods section (subheading “CMV reactivation and SARS-CoV-2 positivity”)

**Reviewer’s comment:**

Was any attempt made to increase comparability of the two cohorts, e.g. propensity score matching accounting for known prognostic factors?

**Authors ‘response:**

We increased comparability between the cohorts by adjusting for key prognostic variables in multivariable models. While propensity score methods are valuable, we chose direct covariate adjustment to preserve sample size and transparency.

Indeed, we didn’t want to decrease our statistical power by loosing patients during the matching process. Moreover, we also wanted to analyze explicitly the effect of the other factors on CMV reactivation and mortality, for example treatment with methylprednisolone.

We agree that propensity score methods (e.g., matching, weighting) are valuable tools to improve comparability between groups. Here, we prioritized covariate adjustment because the number of events allowed for multivariate modeling without risk of overfitting. However, we acknowledge that propensity score techniques could be considered in future sensitivity analyses.

**Reviewer’s comment:**
• Why was the cutoff of four days chosen? Is there any sound rationale for excluding patients based on the cutoff?

**Authors ‘response:**

As previously mentioned, this was the inclusion criteria in the “Premptive treatment for herpesviridae” study (doi.org/10.1186/s13613-020-00793-2) since then applied as a part of routine care in our ICU. The rationale supporting this 4-day delay is based on last decade’s trials investigating CMV reactivation among ICU patients. These trials showed that CMV disease typically occurs within the first 2 weeks of critical illness (reference: Al‑Omari et al. Ann. Intensive Care (2016) 6:110). Notably, the reported incidence of the disease was much higher in studies that screened the patients weekly than those that screened only once within the first 4 days of admission to the ICU, indicating that the disease happens frequently beyond the first 4 days post-admission. Among the studies using PCR detection, the mean and median times of detection ranged from 4 to 12 days after ICU admission, especially in patients undergoing MV which is a known risk factor for CMV reactivation among immunocompetent ICU patients. (Osawa R, Singh N. Cytomegalovirus infection in critically ill patients: a systematic review. Crit Care. 2009;13(3):R68.).

**Reviewer’s comment:**
• In accordance to this potential bias, the SARS-CoV-2-negative group is heterogeneous in terms of admission diagnoses, which may affect the internal validity of comparisons. The authors acknowledge this limitation, but it should be more explicitly discussed in the Discussion section.

**Authors’ response:**

We agree with the reviewer remark that IMV indication in SARS-CoV-2 negative patients varied. However, this heterogeneity increases external validity. Moreover, the majority of patients in both groups developed acute respiratory distress syndrome in which CMV reactivation has been shown to be associated with mortality (Ong et al. ICM 2015 10.1007/s00134-015-4071-z). This limitation was already stated but it has been highlighted in the discussion section of the revised manuscript.

**Reviewer’s comment:**
• CMV serostatus data are missing for more than half of the patients, and there was no standardized protocol for CMV testing. This may lead to underestimation of CMV reactivation incidence and limits the generalizability of results to populations with different CMV seroprevalence.

**Authors’ response:**

We agree that the number of missing data concerning CMV seropositivity at ICU admission doesn’t allow to draw conclusion using this parameter. That was clearly stated in the limits section. However, CMV reactivation was evaluated by blood and/or lung sample PCR and was therefore not influenced by the rate of seropositive patients. CMV screening in blood was in fact protocolized as mentioned above.

**Reviewer’s comment:**
• The authors report, that methylprednisolone was strongly associated with CMV reactivation (SHR 2.84, p<0.001), regardless of SARS-CoV-2 status but dexamethasone was not associated with CMV reactivation. This seems counterintuitive and warrants further explanation.

**Authors’ response:**

We agree with the reviewer that this is a key finding of our study. Indeed, dexamethasone was not associated with CMV reactivation. This finding confirms what was already reported by Luyt et al (doi.org/10.1186/s13613-022-01062-0) who found no association between steroids and CMV reactivation. Here, we wanted to differentiate between the early use of dexamethasone (for COVID-19) and the later use of methylprednisolone (for persistent ARDS). It appears that methylprednisolone, used mainly from the 2^nd^ week of IMV or even later to treat patients with ARDS related lung fibrosis (as described by Meduri et al. doi.org/10.1001/jama.280.2.159.), favored CMV reactivation. The use of methylprednisolone during ARDS is highly debated. Data suggest that late use of methylprednisolone could be associated with poor outcome (Steinberg et al. doi: 10.1056/NEJMoa051693). This can be explained by the risk of increasing secondary infections (bacterial or viral) considering the “ICU related” immunocompromised status. This has been added in the discussion section

**Reviewer’s comment:**
• Antiviral Treatment: The possible protective effect of ganciclovir on mortality is of interest but should be interpreted with caution due to the non-randomized, retrospective nature of the data. The authors appropriately note that this finding should be considered hypothesis-generating for future studies. Also, suggest to cite Naendrup JH, Garcia Borrega J, Eichenauer DA, Shimabukuro-Vornhagen A, Kochanek M, Böll B. Reactivation of EBV and CMV in Severe COVID-19-Epiphenomena or Trigger of Hyperinflammation in Need of Treatment? A Large Case Series of Critically ill Patients. J Intensive Care Med. 2022 Sep;37(9):1152-1158. doi: 10.1177/08850666211053990. Epub 2021 Nov 18. PMID: 34791940; PMCID: PMC9396115.

**Authors’ response:** we acknowledge the reviewer for this interesting reference, in line with our findings. We have added it in the discussion section.

We’d like to thank the reviewer for his helpful comments.

**Reviewer #3:**

**Reviewer’s comment:**

The study analyzes CMV reactivation in patients in a tertiary center, evaluating the risk of reactivation and the factor of exposure to SARS-CoV-2 infection. The methodology included survival analysis with competing risks to assess the risk of CMV reactivation across groups. This analysis is, in fact, recommended. However, a few points remain to be addressed to indicate that it was carried out properly. Furthermore, some uncertainty levels are still missing, especially in Figures 2 and 3. Also, a few issues deserve attention or need clarification.
**Authors’ response:**

We thank the reviewer for this comment and would like to clarify these elements.

Figure 2 is a stacked plot of the events that displays the **cumulative function** of different event types over time. In this plot are represented for each group: the Cumulative incidence of CMV reactivation, Cumulative incidence of extubation without reactivation, Cumulative incidence of death and in the remaining area the censored observations (no event occurred). We think the title of the figure was misleading. We corrected this as follows: « Stacked plots representing cumulative incidence over time of the different events (CMV reactivation, extubation without CMV reactivation, death without CMV reactivation, censoring in the first 60 days post intubation according to SARS-CoV-2 status)”. We hope this will improve clarity.

We also added specifications in the methods section: "Cumulative incidence functions for each event type were estimated using the Aalen-Johansen method to account for competing risks. These functions represent the estimated probability over time of experiencing each event, treating other event types as competing events."

Figure 3 represents the adjusted survival by group of patients after Cox regression model. We also replaced the title as follows: “Adjusted Survival by Group of patients (Cox model adjusted on covariables listed in table 3)”

**Reviewer’s comment:**

Most importantly, it is unclear how the information about patient status regarding CMV exposure prior to hospitalization was obtained. If a patient had a CMV reactivation, I assume it means that this patient had been exposed to CMV prior to hospitalization. However, it should be clear that this comparison involves how patients were assessed for CMV exposure, with reactivation considered only for those with previous exposure.

**Authors’ response:**

Primary infection due to CMV is generally asymptomatic or characterized by the presence of non-specific signs and symptoms and it often occurs during childhood. Approximately 60–80 % of immunocompetent adults are human CMV seropositive (DOI 10.1007/s00134-015-4066-9) [1, 2]. Following primary infection, CMV remains quiescent (latency) and reactivate during immunodepression state, ICU admission and especially mechanical ventilation due to sepsis being a frequently described trigger. Indeed, CMV exposure (*i.e.* seropositivity) would have been of high interest. Unfortunately, this data was missing for more than half patients. We acknowledge this as a limit of our study due to its retrospective design.

**Reviewer’s comment:**
Additionally, the methodology should clearly outline how CMV activation was treated in the analysis, particularly for mortality, which is said to be time-dependent.

**Authors’ response:**

We thank the reviewer for this comment. In our survival analysis, CMV reactivation was modeled as a time-dependent covariate, rather than a baseline (fixed) characteristic. This approach accounts for the fact that CMV reactivation can occur at varying times during follow-up, and it avoids immortal time bias that would result from treating CMV exposure as a fixed covariate.

This is a standard and widely accepted method in time-to-event analyses when dealing with exposures or events that may occur after study entry. Similar methodologies have been extensively described in the survival analysis literature

Technically, we incorporated CMV activation as a time-dependent variable using an extended Cox model, where the covariate status for each individual changes from 0 to 1 at the time of CMV activation. Individuals were considered unexposed until the time of CMV activation and exposed thereafter. This method allows for an accurate estimation of the association between CMV activation and mortality, avoiding immortal time bias. We have clarified this approach in the Methods section of the manuscript.

**Reviewer’s comment:**
The title suggests a comparative study. However, there is no distinct class of studies named "comparative study", and many studies inherently involve some form of comparison. My point is that "comparative study" does not convey the type of study in a title. I suggest rethinking the title.

**Authors’ response:**
Indeed, the title has been modified to better describe the design of the study as recommended in STROBE guidelines.

**Reviewer’s comment:**
The manuscript is well-written, however, it would benefit from some revision on language and style. For instance, in lines 152-153: "so as to distinguish between clinical groups and so to be more informative"

**Authors’ response:**

This sentence has been rewarded and the manuscript extensively reread. Thank you for this remark.

**Reviewer’s comment:**

Abstract: HR=0,4 (use point for decimals). Revise the manuscript for this kind of mistake.

**Authors’ response:**

This has been done, thank you.

**Reviewer’s comment:**
Abstract- Measurements (lines 34-36): The abstract does not mention the use of survival analysis, an important component of the methodology. I recommend including it, even though it might require rewriting the text due to its length. Also, it does not mention primary and secondary endpoints.

**Authors’ response:**

We thank the reviewer for this remark, both suggestions have been done.

**Reviewer’s comment:**

Background (or Introduction section): incidence ranging from 8% to 46% (line 54). Usually, the term incidence has a time component, such as measured over person-years. In this case, it appears to be a proportion of the events, such as the ratio between the number of people with reactivation and the total population of ICU patients or the population that had had CMV in the past. It seems the manuscript refers to the former, but (1) it has to be clear, and (2) this could be the reason for the wide range and should be discussed, although not in this section.

**Authors’ response:**

Indeed, in this sentence, the appropriate term should be “rate”. It has been corrected here and also further in the discussion section, when referring to literature data. In the remainder of the manuscript, the term “incidence” has been conserved as it refers to the number of cases (CMV reactivation) among the ICU population during the study period.

**Reviewer’s comment:**

Background: It appears from lines 69-70 (Refs 10-12) that reactivation has been measured in previous studies. If reactivation has indeed been measured, explain why the statement in 74-75 remains true. In other words, explain what the novelty is in this study.

**Authors’ response:**

We agree with the reviewer’s comment. We performed a careful re-analysis of the literature data available in this field. Several studies have limited effectives (under 100 patients) and their results, both on the incidence of CMV reactivation in SARS-CoV-2 patients and its impact on mortality are conflicting. Using competing risk methods, we did not confirm an association between SARS-CoV2 and CMV but we showed a negative effect on mortality of viral co-infection. The main originality of our cohort, apart from its size, stays in the fact that we put in light the role of methylprednisolone (and not dexamethasone) as a risk factor for CMV reactivation and a lower mortality in patients that were treated with ganciclovir. We placed our findings in the context of existing literature and what our study adds to the field in the introduction but especially in the discussion sections.

**Reviewer’s comment:**
Methodology:
How was CMV activation treated as a time-dependent variable in the mortality analysis? I recommend making it clear.

**Authors’ response:**

As mentioned above, we have added the following specifications into the methodology section: “CMV reactivation was considered as a time dependent variable in the mortality analysis (the covariate status for each individual changes from 0 to 1 at the time of CMV activation eg individuals were considered unexposed until the time of CMV activation and exposed thereafter)..”

**Reviewer’s comment:**

Line 147: Please clarify what it means that the SARS-CoV-2 positive variable was forced into the analysis.

**Authors’ response:**

By stating that the SARS-CoV-2 positivity variable was “forced into the analysis,” we meant that this variable was included in the multivariate model regardless of its statistical significance in univariate screening. In other words, it was retained a priori, based on its clinical relevance and central role in the study question, and not selected or excluded through automated model selection procedures.

This approach ensures that the effect of SARS-CoV-2 infection on the outcome is always estimated, even if other covariates might have stronger statistical associations. We have clarified this point in the revised Methods section for greater transparency.

**Reviewer’s comment:**
Some colors used in Figures 2 and 3 are difficult to distinguish. I recommend using a more distinguishable set of colors.

**Authors’ response:**
We are sorry but we tried several set of colors and the result was comparable to the initial version.We decided therefore to conserve it.

**Reviewer’s comment:**
In Figures 2 and 3, the uncertainty levels should be included.

**Authors’ response:**

For figure 2 and 3, we have added precisions in the methods section azs mentioned above to better explain them. We also changed the titles of figures for more clarity.

**Reviewer’s comment:**
For Figure 2, how was probability computed? It seems that it was the proportion of individuals with the signaled events over time. If so, I would rather say proportion of frequency instead of probability.

**Authors’ response:**

We thank the reviewer for this observation. In Figure 2, the cumulative incidence functions (CIFs) for each of the four mutually exclusive outcomes (CMV reactivation, extubation without reactivation, death, and censoring) were estimated using a competing risks framework. The probabilities shown correspond to nonparametric estimates of the cumulative incidence of each event over time, accounting for the presence of competing events. While these estimates are indeed based on observed event proportions, they represent cumulative probabilities in the statistical sense, derived from the Aalen-Johansen estimator, which generalizes the Kaplan-Meier estimator for competing risks.

These curves can be named as "cumulative incidence probabilities" which is more precise than simply “proportions.” We have clarified the terminology in the figure legend and in the Methods section to avoid ambiguity.

**Reviewer’s comment:**

For Figure 2, the caption defines T as time in days. I strongly recommend changing the label of the x-axis to express this information.

**Authors’ response:**

This has been done, thank you.

**Reviewer’s comment:**
Other minor issues:

Line 85: correct "all patients form…".

**Authors’ response:**

This has been done, sorry for the mistake.

**Reviewer’s comment:**

Line 128: "if not the" - wording sounds strange.

**Authors’ response:**

This has been done, thank you.

We’d like to thank the reviewer for his helpful comments.

6. PLOS authors have the option to publish the peer review history of their article ([what does this mean?](https://urldefense.com/v3/__https:/track.editorialmanager.com/CL0/https:*2F*2Fjournals.plos.org*2Fplosone*2Fs*2Feditorial-and-peer-review-process*23loc-peer-review-history/1/010f01986054b280-82693657-4a3d-462a-8e7f-e1df07129cd1-000000/QhjDuLCqTz_f5racX1FUOU6goz2OtQCDGBo3Vt-9aEI=222__;JSUlJSUl!!JQ5agg!ZuTmpheA5EvGYYXLom0TNFeVPgbWmj4p3xQmpoNQifisfAMHACt_vpjaXYHJfXImfEasx8pSmyaQJ-BrQ4RZ$)). If published, this will include your full peer review and any attached files.

If you choose “no”, your identity will remain anonymous but your review may still be made public.

**Do you want your identity to be public for this peer review?** For information about this choice, including consent withdrawal, please see our [Privacy Policy](https://urldefense.com/v3/__https:/track.editorialmanager.com/CL0/https:*2F*2Fwww.plos.org*2Fprivacy-policy/1/010f01986054b280-82693657-4a3d-462a-8e7f-e1df07129cd1-000000/xO9hqrtWs_Qn8s8aKulWUbPMMJatp4FLy37K3tD9rIU=222__;JSUl!!JQ5agg!ZuTmpheA5EvGYYXLom0TNFeVPgbWmj4p3xQmpoNQifisfAMHACt_vpjaXYHJfXImfEasx8pSmyaQJw6aCFoa$).

Reviewer #1: No

Reviewer #2: No

Reviewer #3: No
