# Supplementary material for: Cytomegalovirus reactivation in mechanically ventilated patients with or without SARS-CoV-2 infection: A retrospective cohort study
Source: PLoS One. 2025 Oct 27;20(10):e0328494. doi: 10.1371/journal.pone.0328494 (PMC12558532; doi:10.1371/journal.pone.0328494)
Supplement: Attachment — Submitted filename: Responses to Editor and Reviewers.docx [file pone.0328494.s004.docx]

Dear Editor and Reviewers,

Thank you for your comments and for offering us the possibility to submit a revised version of our manuscript.

Please find enclosed our responses to the points you raised.

**Additional Editor Comments:**

**Editor’s comment:**

The manuscript has undergone significant improvement, and two of the three reviewers are now satisfied with the changes.

**Authors’ response:**

We thank the Editor for this positive appreciation.

**Editor’s comment:**

One reviewer, however, has raised a remaining point that I believe should be addressed to strengthen the work.

Specifically, the reviewer notes that information about CMV status at admission was only available for half of the patients. Given that the manuscript focuses on CMV reactivation, the reviewer recommends that you provide additional consideration of this limitation. While I understand it may not be possible to resolve fully due to the retrospective nature of the study, I ask that you please:

1. **Clarify and expand the discussion** regarding the potential biases introduced by missing CMV serostatus at admission.

**Author’s response:**

**We understand the importance of such a question. Therefore we decided to perform a complementary analysis to evaluate if the lack of serological status for about half of our population might have represented an important bias in our results.**

**CMV serological status at ICU admission was available for 166 (50.3%) patients. There was no difference between the two groups: 75/147 (51.0%) of SARS-CoV-2 negative patients had a known CMV serological status vs. 91/183 (49.7%) for the SARS-CoV-2 positive patients (p=0.81). Among patients with a known CMV serological status, 129 (77.8%) were** **seropositive. This result is in line with what is usually described: 60-80% of adults are CMV seropositive in western countries and it has been shown that** **seroprevalence gradually increases from 50% in young adults to 90% in the elderly (*Papazian et al. Intensive Care Med (2016) 42:28–37 DOI 10.1007/s00134-015-4066-9; Ong et al. DOI: 10.1097/CCM.0000000000000712*). Considering the mean age of our population, such a seroprevalence is not surprising. When focusing on the patients for which the serological status was known, 71 (78%) SARS-CoV-2 positive patients were seropositive and 58 (77%) SARS CoV-2 negative patients were seropositive (p=0.95). We think that the equivalent repartition of seropositive patients among COVID-19 and non-COVID-19 patients is rather reassuring towards the risk of bias. These data have been added in the methods section and in the results section, in the text and in the table 1.**

**To confirm our results, we also performed sensitivity analyses focusing only on patients for which serological status was known and who were seropositive, therefore that could effectively reactivate CMV during their ICU stay. The results of these complementary analyses have been added in the supplementary material (Tables S1 and S2). Concerning CMV reactivation, methylprednisolone use remained a significant risk factor in this sub-population (129 patients). Time to intubation did not reach significance anymore (p=0.06) probably because of a lack of statistical power. Concerning mortality risk factors, in this sub-population, SARS-CoV-2 and CMV reactivation did not reach significance any more (p= 0.06 and 0.09 respectively) probably because of a loss of statistical power.**

**Lastly, the discussion has been modified according to these elements. Serological status is now clearly addressed in the discussion section in which we acknowledge the limits of missing data and bring in light the elements that comfort our results despite this limitation.**

**Overall, considering the equivalent repartition of CMV known status and CMV seropositivity in both groups with a high prevalence of CMV seropositivity, we remain confident about the results in our whole cohort.**

**Editor’s comment:**

**Revise Table 1 (if feasible)** to include the number of patients with known CMV status, stratified by SARS-CoV-2 positive and negative groups.

**Authors’response:**

This has been done as requested.

**Editor’s comment:**

 Moreover:

- **Revisit the discussion of corticosteroid use (methylprednisolone vs. dexamethasone):** this point comes across more clearly in your response to reviewers than in the main text.

**Authors’ response:**

Following the Editor advice, we have clarified and developed this point of the discussion concerning the effects of methylprednisolone but not dexamethasone on CMV reactivation putting it in light the still ongoing debate on benefits and risks of steroids in ARDS patients.

**Editor’s comment:**

- **Address language/formatting issues:** please carefully proofread the newly added text to correct typos,  improve clarity and remove redundancies.

**Authors’ response:**

The manuscript has been carefully reread in this sense.

**Editor’s comment:**

 I do not anticipate that additional analyses will be required if a careful discussion is provided; however, if you are able to perform a sensitivity analysis restricted to patients with known CMV status, that would further strengthen the manuscript.

**Authors’ response:**

These sensitivity analyses have been done and added in the supplementary material.

Please submit a revised version with a short response letter indicating how you have addressed these points.

[Note: HTML markup is below. Please do not edit.]

Reviewers' comments:

Reviewer's Responses to Questions

**Comments to the Author**

1. If the authors have adequately addressed your comments raised in a previous round of review and you feel that this manuscript is now acceptable for publication, you may indicate that here to bypass the “Comments to the Author” section, enter your conflict of interest statement in the “Confidential to Editor” section, and submit your "Accept" recommendation.

Reviewer #1: All comments have been addressed

Reviewer #2: All comments have been addressed

Reviewer #3: (No Response)

2. Is the manuscript technically sound, and do the data support the conclusions?

The manuscript must describe a technically sound piece of scientific research with data that supports the conclusions. Experiments must have been conducted rigorously, with appropriate controls, replication, and sample sizes. The conclusions must be drawn appropriately based on the data presented.

Reviewer #1: Yes

Reviewer #2: Yes

Reviewer #3: Partly

3. Has the statistical analysis been performed appropriately and rigorously?

Reviewer #1: Yes

Reviewer #2: Yes

Reviewer #3: No

4. Have the authors made all data underlying the findings in their manuscript fully available?

The [PLOS Data policy](https://urldefense.com/v3/__http:/track.editorialmanager.com/CL0/http:*2F*2Fwww.plosone.org*2Fstatic*2Fpolicies.action*23sharing/1/010f0198f50dbf7b-f1312fb4-06da-455c-853e-3a3f5e4b18f7-000000/Yx-kjus4OMS3L8SVgW3QjlahgqvqrqWbX-t6JaScT6M=226__;JSUlJSU!!JQ5agg!bkPAojznd6GYhotPJf0LUKvl5YN60NN8g0ZWrIjaZn2MQf5AUVvUvdezw5kORfVR_JCfLq2v3_GWKmR-qLVf$) requires authors to make all data underlying the findings described in their manuscript fully available without restriction, with rare exception (please refer to the Data Availability Statement in the manuscript PDF file). The data should be provided as part of the manuscript or its supporting information, or deposited to a public repository. For example, in addition to summary statistics, the data points behind means, medians and variance measures should be available. If there are restrictions on publicly sharing data—e.g. participant privacy or use of data from a third party—those must be specified.

Reviewer #1: Yes

Reviewer #2: Yes

Reviewer #3: Yes

5. Is the manuscript presented in an intelligible fashion and written in standard English?

PLOS ONE does not copyedit accepted manuscripts, so the language in submitted articles must be clear, correct, and unambiguous. Any typographical or grammatical errors should be corrected at revision, so please note any specific errors here.

Reviewer #1: Yes

Reviewer #2: Yes

Reviewer #3: Yes

6. Review Comments to the Author

Please use the space provided to explain your answers to the questions above. You may also include additional comments for the author, including concerns about dual publication, research ethics, or publication ethics. (Please upload your review as an attachment if it exceeds 20,000 characters)

**Reviewer #1:**

**Reviewer’s comment:**

The authors should be commended for their nice study and thorough responses to the reviewers. I have no additional concerns.

**Authors’response:**

We thank the reviewer for his/her comment and positive appreciation of our work.

**Reviewer #2:**

**Reviewer’s comment:**

Please exclude patient IDs from the data as standard recomendations in order to protect patients. Otherwise very good revision, thank you!

**Authors’ response:**

Patients’ identification numbers have been deleted from the primary data. We thank the reviewer for his/her comment and positive appreciation of our work.

**Reviewer #3:**

**Reviewer’s comment:**

The manuscript underwent significant improvement - however, in light of the fact that information about CMV status at admission was available for only half of the patients, I believe that more should be described about CMV status at admission. For instance, Table 1 can include n of CMV status positive and CMV status for SARS-CoV-2 positive and negative patients.

**Authors’ response:**

We agree with the reviewer about the importance of CMV status when evaluating CMV reactivation. Therefore, we analyzed the subpopulation for whom the serological status for CMV was known at ICU admission.

**CMV serological status at ICU admission was available for 166 (50.3%) patients. There was no difference between the two groups: 75/147 (51.0%) of SARS-CoV-2 negative patients had a known CMV serological status vs. 91/183 (49.7%) for the SARS-CoV-2 positive patients (p=0.81). Among patients with a known CMV serological status, 129 (77.8%) were seropositive. This result is in line with what is usually described: 60-80% of adults are CMV seropositive in western countries and it has been shown that** **seroprevalence gradually increases from 50% in young adults to 90% in the elderly (*Papazian et al. Intensive Care Med (2016) 42:28–37 DOI 10.1007/s00134-015-4066-9; Ong et al. DOI: 10.1097/CCM.0000000000000712*). Considering the mean age of our population, such a seroprevalence is not surprising. When focusing on the patients for which the serological status was known, 71 (78%) SARS-CoV-2 positive patients were seropositive and 58 (77%) SARS CoV-2 negative patients were seropositive (p=0.95). We think that the equivalent repartition of seropositive patients among COVID-19 and non-COVID-19 patients is rather reassuring towards the risk of bias. These data have been added in the methods section and in the results section, in the text and in the table 1.**

**Reviewer’s comment:**
Since CMV status was not part of the inclusion/exclusion criteria, the study had alternatives for the survival analysis: (1) include only individuals with CMV status - in this case, losing statistical power and incurring a bias failing to consider several potential CMV exposed individuals; and (2) include all individuals - in this case many individuals would not experience reactivation because were not exposed, which is a different bias.
I recommend evaluating the results with additional analysis using a subset that had CMV status at admission and comparing the effects in both analyses.

**Authors’ response:**

According to the reviewer’s recommendation, to confirm our results, we performed sensitivity analyses focusing only on patients for which serological status was known and who were seropositive (reactivation among seronegative patients during the ICU stay being highly unlikely), therefore that could effectively reactive CMV during their ICU stay.

**The results of these complementary analyses have been added in the supplementary material (Tables S1 and S2). Concerning CMV reactivation, methylprednisolone use remained a significant risk factor in this sub-population (129 patients). Time to intubation did not reach significance anymore (p=0.06) probably because of a lack of statistical power. Concerning mortality risk factors, in this sub-population, SARS-CoV-2 and CMV reactivation did not reach significance any more (p= 0.06 and 0.09 respectively) probably because of a loss of statistical power.**

**Considering the equivalent repartition of CMV known status and CMV seropositivity in both groups with a high prevalence of CMV seropositivity, we remain confident about the results in our whole cohort and chose to maintain in the main manuscript.**

**Reviewer’s comment:**

Then, a necessary discussion will be warranted with these options and the results, rather than stating that CMV serological status was missing for half the population and therefore not shown. In fact, the manuscript is about CMV reactivation, and this evaluation is important to comprehend the potential biases.

**Authors’response:**

We agree. The discussion has been modified according to these elements. Serological status is now clearly addressed in the discussion section in which we acknowledge the limits of missing data and bring in light the elements that comfort our results despite this limitation.

**Reviewer’s comment:**
Line 354-355 "freeing us from the bias of changing standards of care"- wording sounds strange, I recommend revising it.

**Authors’response:**

Indeed, this sentence has been reworded.

**Reviewer’s comment:**
Choice of colors in Figures: I do think it was possible to change the colors in Figures; but this was an optional recommendation.

**Authors’ response:**

We thank the reviewer for his/her understanding.

**Reviewer’s comment:**

Line : "CMV serological status was missing for more than half of our population which is why we chose not to display these
data" - I do not agree. It is actually one more reason to present this data, as this variable is important.

**Authors’response:**

This has been modified as described upward.

**Reviewer’s comment:**
Correct "Student T test": Student's t-test.

**Authors’response :**

This has been done.

We thank the reviewer for his/her highly relevant comments.
